# The effect of surface wettability on water vapor condensation in nanoscale

**DOI:** 10.1038/srep19192

**Published:** 2016-01-12

**Authors:** D. Niu, G. H. Tang

**Affiliations:** 1 MOE Key Laboratory of Thermo-Fluid Science and Engineering, School of Energy and Power Engineering, Xi’an Jiaotong University, Xi’an 710049, China

## Abstract

The effect of surface wettability on condensation heat transfer in a nanochannel is studied with the molecular dynamics simulations. Different from the conventional size, the results show that the filmwise mode leads to more efficient heat transfer than the dropwise mode, which is attributed to a lower interfacial thermal resistance between the hydrophilic surface and the condensed water compared with the hydrophobic case. The observed temperature jump at the solid-liquid surface confirms that the hydrophilic properties of the solid surface can suppress the interfacial thermal resistance and improve the condensation heat transfer performance effectively.

Advanced enhancement technologies of condensation heat transfer have the potential to significantly improve thermal efficiency and reduce the cost of energy consumption in many applications such as the electricity, water desalination and chemical engineering. In particular, it has been confirmed that the dropwise condensation is able to produce heat transfer coefficient an order of magnitude higher than the filmwise condensation^1^,^2^,^3^,^4^. Furthermore, the mode of condensation mainly depends on the surface wettability. So designing stable and reliable surface that enables dropwise condensation is particularly meaningful^5^,^6^,^7^. All the existing work is based on the premise that the dropwise mode is more efficient than the filmwise mode in the condensation heat transfer in macroscale. However, the condensation heat transfer characteristics in nanoscale or in a nanochannel remain unclear. As we all know, the interface effect in nanoscale plays a significant role in heat transfer^8^, especially with the rise and applications of nanocomposite materials^9^,^10^. We need to pay more attention to investigate the heat transfer characteristics in nanoscale because of a high specific surface area of such new materials. Therefore, for the condensation heat transfer in nanoscale, it is indispensable to take the solid-liquid interfacial thermal resistance also known as the Kapitza resistance into account. Current theoretical understanding of interfacial thermal resistance is mainly concentrated in the acoustic mismatch model (AMM) and the diffusive mismatch model (DMM)^11^. For the acoustic mismatch model, it assumes that the Kapitza resistance is induced by the mismatch in acoustic impedance of two neighboring materials. However, in the diffusive mismatch model, it is assumed that the phonons are scattered at the interface and the resistance is determined by the mismatch in vibrational density of states (VDOS) of two adjacent materials at the interface.

Both theoretical^12^,^13^ and molecular dynamics simulation results^14^,^15^ have proved that the interface wettability is the key to affect interfacial thermal resistance. In particular, Wang and Keblinski^16^ determined the thermal resistance of a liquid-solid interface with and without nanoscale roughness and revealed that the key factor controlling interfacial thermal resistance is the strength of the bonding between liquid and solid atoms. Hu and Sun^17^ examined the effect of width-to-spacing ratio of nanopatterns on interfacial thermal resistance of water boiling on a gold surface using molecular dynamics simulations. The simulations revealed that the increase of the height of nanopatterns leads to a reduction in the Kapitza resistance by increasing the interaction energy per unit area and would help to improve boiling heat transfer on nanopatterned surface. Murad and Puri^18^ performed molecular dynamics simulations to study the thermal transport across nanoscale solid-fluid interfaces. They found that the interfacial thermal resistance could be decreased by increasing the fluid pressure or by adsorbing additional fluid molecule layers with more hydrophilic surface. The unsteady nanoscale thermal transport at Ar-Fe interface was simulated using non-equilibrium molecular dynamics simulations^19^. The simulation results showed that the temperature discontinuities between the solidlike interfaces and their neighboring fluid molecules are caused by vacancies at the migration sites adjacent to the walls.

In this study, we investigate the interface effect on the condensation phase change in a nanochannel using the molecular dynamics simulations, which has not been reported in the literature. Depending on the wettability of the condensation surface, the condensation exhibits dropwise or filmwise mode just as in macroscale. However, we find that the filmwise mode shows more efficient heat transfer than the dropwise mode in nanoscale because the hydrophilicity of the condensation surface can reduce the interfacial thermal resistance and improve the heat transfer performance effectively.

## Results and Discussion

**Figure 1:**
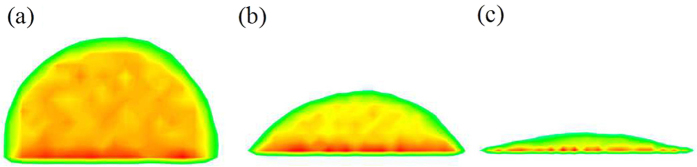
Density contours of water droplets for different surface wettability. (**a**) *ε*_water-Pt_ = 0.01 eV, (**b**) *ε*_water-Pt_ = 0.015 eV and (**c**) *ε*_water-Pt_ = 0.02 eV.

**Figure 2:**
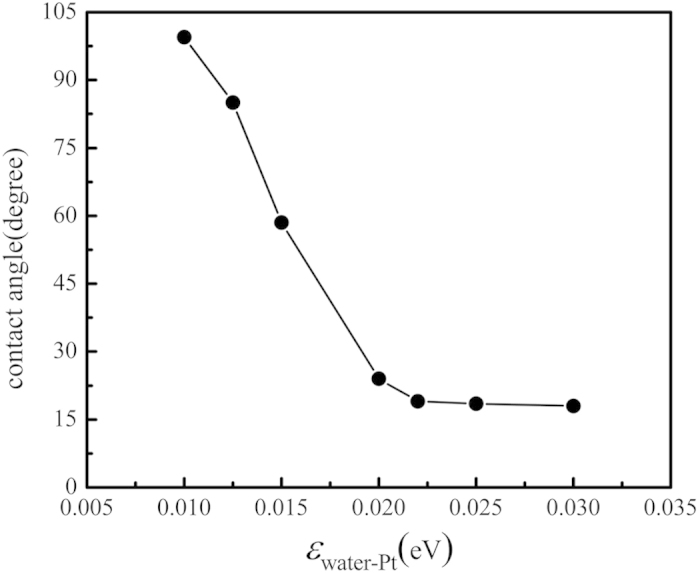
Contact angles for different surface wettability.

To confirm water contact angle from the density field, we define the droplet boundary as the contour line with 0.5 g/cm^3^ in the density field. Droplet on the smooth solid surface as a reference of the wettability is simulated and the results are shown in Fig. 1. Obviously, the hydrophilic surface attracts water molecules strongly than the hydrophobic surface as the solid-water characteristic energy increases. The range of 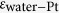 from 0.01 eV to 0.03 eV is able to cover the surface wettability from hydrophobic to hydrophilic. Actually, when 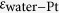 increases to 0.02 eV, the solid surface has already presented a perfect hydrophilicity as shown in Fig. 1c. The approximate contact angles obtained from the present simulation are shown in Fig. 2.

**Figure 3:**
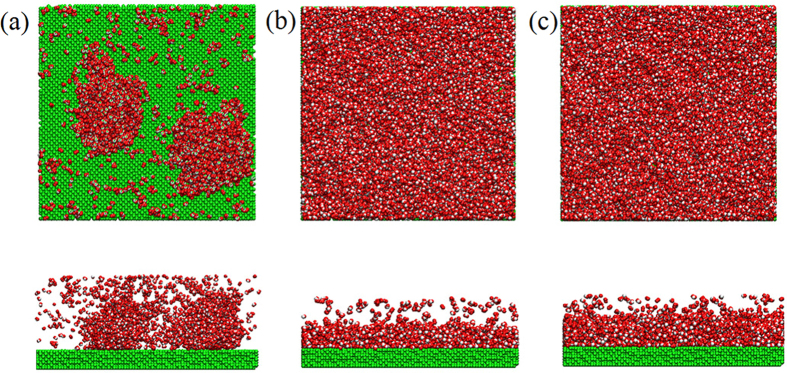
Condensation mode on the cold surface with different wettability. (**a**) 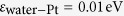, (**b**) 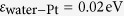, (**c**) 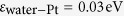.

**Figure 4:**
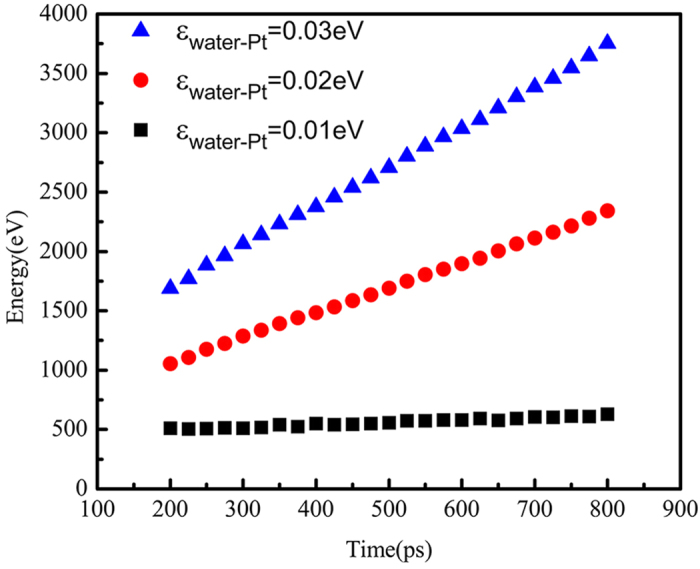
Total heat transfer accumulation with the time for surfaces with different wettability.

The present nanoscale condensation exhibits similar dropwise mode or filmwise mode based on the surface wettability as in macroscale. Figure 3 shows different condensation modes of the water vapor when the energy parameter changes from 0.01 eV to 0.03 eV. In Fig. 3a, the solid surface is hydrophobic at which the energy parameter between the solid surface and the water is 0.01 eV. We can observe that the nano-droplet forms on the cold surface. Compared with Fig. 3a, the liquid film covers the whole surface due to the well wettability of the cold wall in both Fig. 3b,c. We also calculate the heat exchange between the water vapor and the cold wall. As shown in Fig. 4, the accumulation of heat transfer is calculated for three cold walls with different surface wettability and all the three curves show good linearity, which illustrates that the heat flux defined as the ratio of the slope of each curve to the cold surface area is constant during condensation process and is almost not affected by the distribution of water particles. We find that the hydrophobic surface does not show advantage in heat transfer enhancement as in macroscale. Instead, the solid surfaces with hydrophilic property show a higher heat transfer performance.

**Figure 5:**
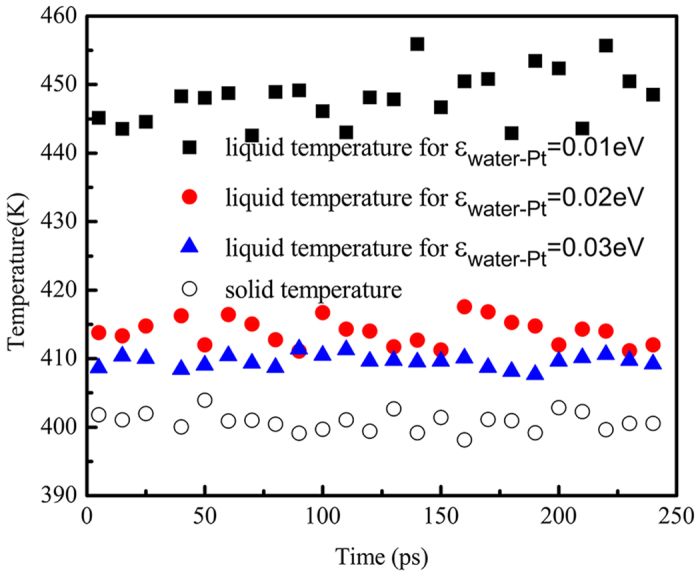
Temperature jump between solid surface and liquid for different surface wettability.

From the simulation results we can see that the condensation in nanoscale exhibits different heat transfer characteristics from the one in macroscale. Considering the heat transfer process from the hot wall to the cold wall, we just change the surface wettability of the cold surface. Thus, the surface wettability is the main factor that affects the condensation heat transfer in nanoscale. We also calculate the temperature distribution close to the solid-liquid interface for different surface wettability. As shown in Fig. 5, there exists a relatively large temperature jump between the hydrophobic smooth surface and the condensate, which indicates that the hydrophobicity of the cold wall results in a significant interfacial thermal resistance. On the contrary, the hydrophilic solid surface can effectively suppress the interfacial thermal resistance and improve the heat transfer performance.

In summary, the condensation mode and condensation heat transfer in a nanochannel with different surface wettability has been investigated using the molecular dynamics simulations. The condensation exhibits dropwise or filmwise mode depending on the wettability of the cold surface as in macroscale. Abnormally, the dropwise mode in nanoscale does not show more efficient heat transfer than the filmwise mode. A large temperature jump is observed between the liquid and the hydrophobic solid surface, which causes the dropwise condensation to lose its advantage in condensation heat transfer enhancement. Instead, the hydrophilicity of the solid surface can reduce the interfacial thermal resistance and improve the heat transfer performance effectively. The present study shows that, for the first time, the interfacial effect is the most dominant in nanoscale condensation heat transfer, which is totally different from the conventional size.

## Methods

All the present simulations are performed based on the LAMMPS package^20^. In this study, the embedded-atom method (EAM)^21^ is applied for the interatomic forces of Pt-like atoms. The TIP3P^22^ model that has a single Lennard-Jones center representing an oxygen atom together with three charges, −0.834 e for the O atom and +0.417 e for the H atoms with an angle of 104.52˚ between the atoms is employed to describe the water-water interactions. For this choice of EAM potential, its functional form is


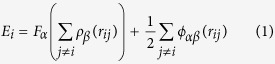


where *F* is the embedding energy as a function of the atomic electron density *ρ*, *ϕ* is a pair potential interaction, and *α* and *β* are the element types of atoms *i* and *j*, respectively. The multi-body nature of the EAM potential is a result of the embedding energy term. For the TIP3P water model, the Van der Waals interactions and the Coulomb interactions are calculated with


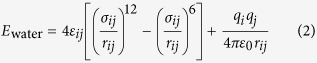


where *ε* and *σ* are the characteristic surface energy and the van der Waals radius, respectively. *q*_*i*_ and *q*_*j*_ are the partial atomic charge of atom *i* and *j*, respectively, and *ε*_0_ is the vacuum permittivity. The cutoff distance of 12 Å is used for the 12-6 LJ potential and short-range Coulombic interactions. The particle-particle particle-mesh (PPPM) method is adopted to compute the long-range Coulombic forces. The energy and distance parameters are 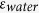 = 0.0044 eV and 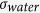 = 3.188 Å for oxygen atoms, respectively. The 12-6 LJ potential is also employed to describe the interaction between the Pt-like solid wall and the water molecules. Energy parameter 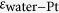 changes in the range of 0.01–0.03 eV to cover the surface property from the hydrophobic to the hydrophilic.

**Figure 6:**
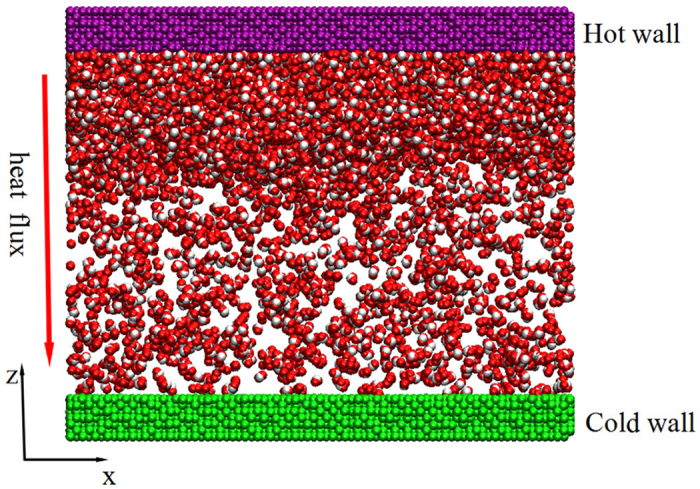
Simulation setups of water-Pt systems.

The system setup for the non-equilibrium molecular dynamics (NEMD) simulations of water-Pt system is schematic in Fig. 6, which contains 17693 water molecules and 44800 Pt-like atoms. Periodic boundary conditions are applied in all the three directions. Figure 6 presents an initial snapshot before the condensation occurs and the liquid film is concentrated on the hot wall as a vapor source just like in the macroscale condensation experiment. For the cold and hot walls, the positions of the outside layer of the Pt-like atoms are kept fixed to avoid the deformation of the solid wall. The next two layers of the Pt-like atoms are treated as heating source or cooling source corresponding to the hot wall and cold wall. The inside four layers of atoms as Pt-like solid wall are built to exchange energy between the water and the heating source or cooling source. Such a setting was adopted to build the heating or cooling surface in previous studies^23^,^24^,^25^ and has been proved to effectively avoid artificial thermal resistance caused by the applied thermostat^26^. In all cases, we just change the interaction parameters between the cold wall and the water to simulate condensation surface with different surface wettability. All the simulations are performed in two stages and a time step of 5 fs is adopted in all cases. The Pt-water system is firstly equilibrated in an NVT (N is the number of atoms, V is the volume, and T is the temperature) ensemble at 500 K, and then the structure after equilibrium as shown in Fig. 6 is used as the initial configuration before the condensation occurs. To create a temperature difference, the thermostat applied on the water molecules is removed and the NVE (E is the total energy) ensemble is used for the water. The temperature of the cold wall is reduced to 400 K controlled by the Nose-Hoover thermostat, while the hot wall is always maintained at 500 K. Then, the high temperature water vapor begins to condensate on the cold surface.

**Figure 7:**
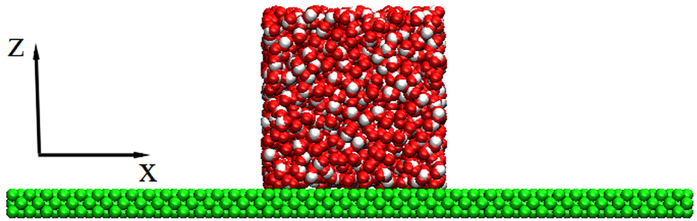
Initial water box on the Pt-like surface.

To confirm the wettability of the solid surface, we refer to the density contour of the droplet^27^,^28^ to determine the contact angle of a nano-droplet on Pt-like solid surface and then we can obtain the wettability of different surfaces qualitatively. The initial water cubic box containing 1981 water molecules is placed on the Pt-like solid surface as shown in Fig. 7. Based on different surface wettability, the initial water box will evolve into a droplet with different contact angles. In the simulation, the Pt-like solid wall is treated as a rigid body and there is no relative movement between Pt-like atoms. The water molecular model and parameters are consistent with the above. The simulation is carried out for 1 ns. In the first 500 ps, the initial water box forms into a droplet on the solid surface gradually and the height of the droplet mass center in the *z* direction tends to be almost unchanged. We collect the coordinates of each molecule and finally take the time average to obtain the density contour of the droplet in the following 500 ps.

## Additional Information

**How to cite this article**: Niu, D. and Tang, G. H. The effect of surface wettability on water vapor condensation in nanoscale. *Sci. Rep.* **6**, 19192; doi: 10.1038/srep19192 (2016).
